# An extranodal Richter’s syndrome presenting with cardiac diffuse large B-cell lymphoma: a case report

**DOI:** 10.1186/s12872-023-03663-4

**Published:** 2023-12-21

**Authors:** Hua Wang, Wei-hong Yan, Jun-jie Sun, Mei Dong, Nan Zhang, Tong Liu, Nian-peng Song, Lin Zhong

**Affiliations:** 1 Department of Cardiology, Yantai Yuhuangding Hospital, Qingdao Medical College, Qingdao University, No. 20 East Yuhuangding Road, Yantai, 264000 China; 2 Department of Ultrasound, Yantai Yuhuangding Hospital, Qingdao Medical College, Qingdao University, Yantai, China; 3 Tianjin Key Laboratory of Ionic-Molecular Function of Cardiovascular Disease, Department of Cardiology, Tianjin Institute of Cardiology, Second Hospital of Tianjin Medical University, Tianjin, China

**Keywords:** Cardiac tumor, Cardiac lymphoma, Richter's syndrome, Contrast-enhanced echocardiagraphy

## Abstract

**Background:**

Richter’s syndrome (RS) defines the transformation of chronic lymphocytic leukemia into high-grade lymphoma, which usually involves lymph nodes and bone marrow. Extranodal involvement of the heart is an extremely rare condition. Patients with heart involvement tended to have a low response to chemotherapy and relative poor prognosis. The transformation process of RS is often insidious and nonspecific making it challenging to diagnose.

**Case presentation:**

A 64-year-old woman wih a history of chronic lymphocytic leukemia (CLL) presented with intermittent chest pain and was diagnosed with non-ST-elevation myocardial infarction (NSTEMI). However, the contrast enhanced echocardiography revealed a large irregular mass, measuring about 75.4 mm × 37.5 mm, located on the lateral and posterior wall of the right ventricle. Biopsy of the cardiac mass and the results revealed diffuse large B-cell lymphoma.

**Conclusions:**

We present a case of a 64-year-old woman with aggressive diffuse large B-cell lymphoma involving the heart. This case could provide some insights in the diagnosis of cardiac lymphoma.

**Supplementary Information:**

The online version contains supplementary material available at 10.1186/s12872-023-03663-4.

## Background

Richter's syndrome (RS) is an uncommon entity that describes the transformation of chronic lymphocytic leukemia (CLL) or small lymphocytic lymphoma (SLL) into a more aggressive type of lymphoma, which presents as two different pathologic entities: large B-cell lymphoma (DLBCL) or Hodgkin's lymphoma (HL) [1]. The development of lymphoma in CLL setting was originally reported by Maurice N. Richter in 1928 [2], and the term “Richter's syndrome” was first described in 1964 by Lortholary et al*.* to define the development of malignant reticulopathy in 14 patients with CLL [1]. This pattern of transformation has been reported in 2% to 10% of patients with CLL/SLL [3]. Its locations and clinical manifestations are varied. However, the Cardiac involvement has rarely been reported. The RS transformation process is insidious and nonspecific, and it is often difficult to diagnose**.** Here, we report a case of RS presented with cardiac diffuse large B-cell lymphoma in a patient with an 8-year history of CLL.

## Case presentation

**Fig. 1:**
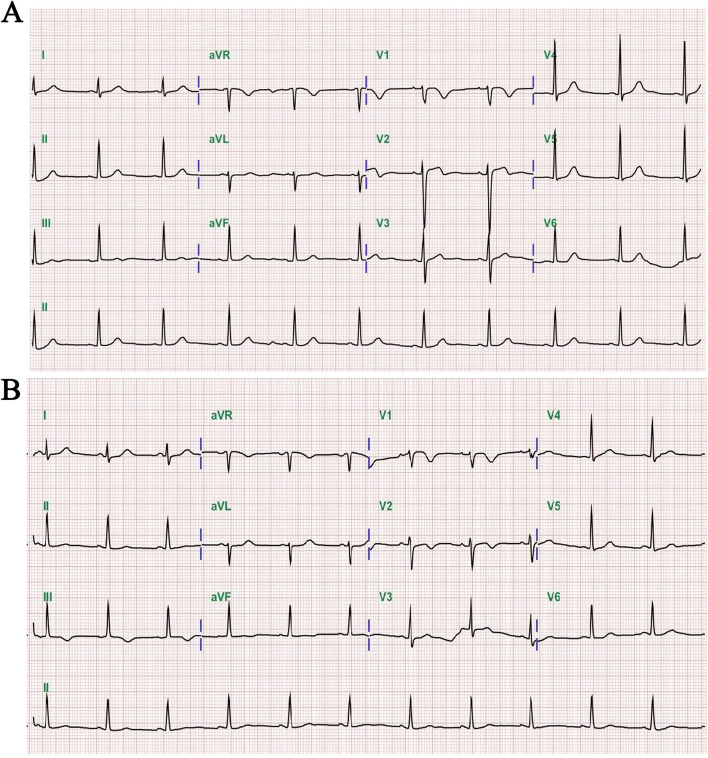
The electrocardiogram (ECG) shows inverted T wave in inferior leads and leads V1, V2. **A** ECG on the first admission; (**B**) ECG on the second admission one month later

**Fig. 2:**
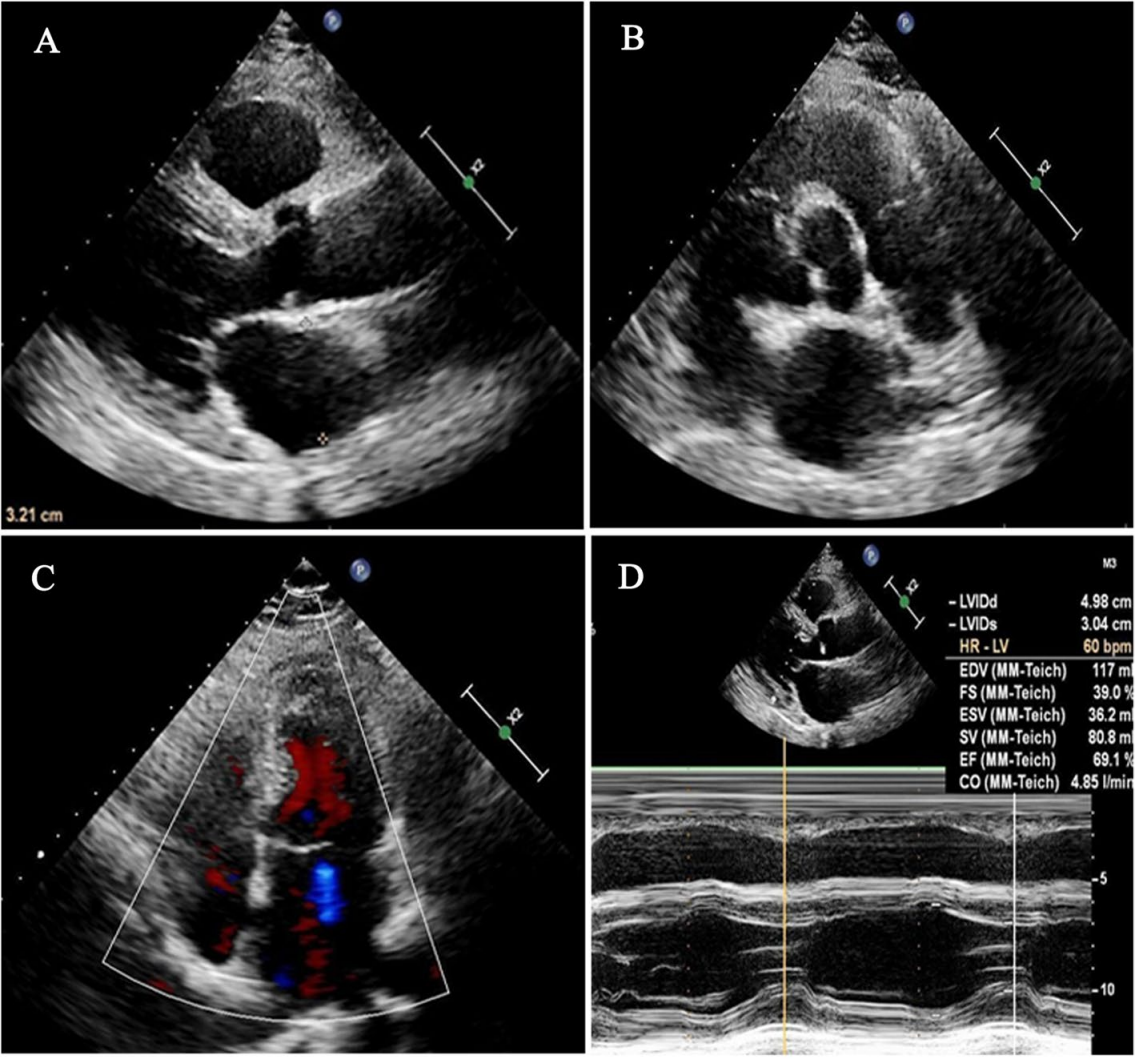
Echocardiographic findings on the first admission indicated the heart chamber size, structure, and systolic function were normal except for mild mitral regurgitation. **A** Parasternal long axis view and (**B**) Parasternal short axis view showed normal heart chamber size. **C** Apical 4 chamber view showed mild mitral regurgitation. **D** M-mode echocardiography indicated normal cardiac systolicfunction

**Fig. 3:**
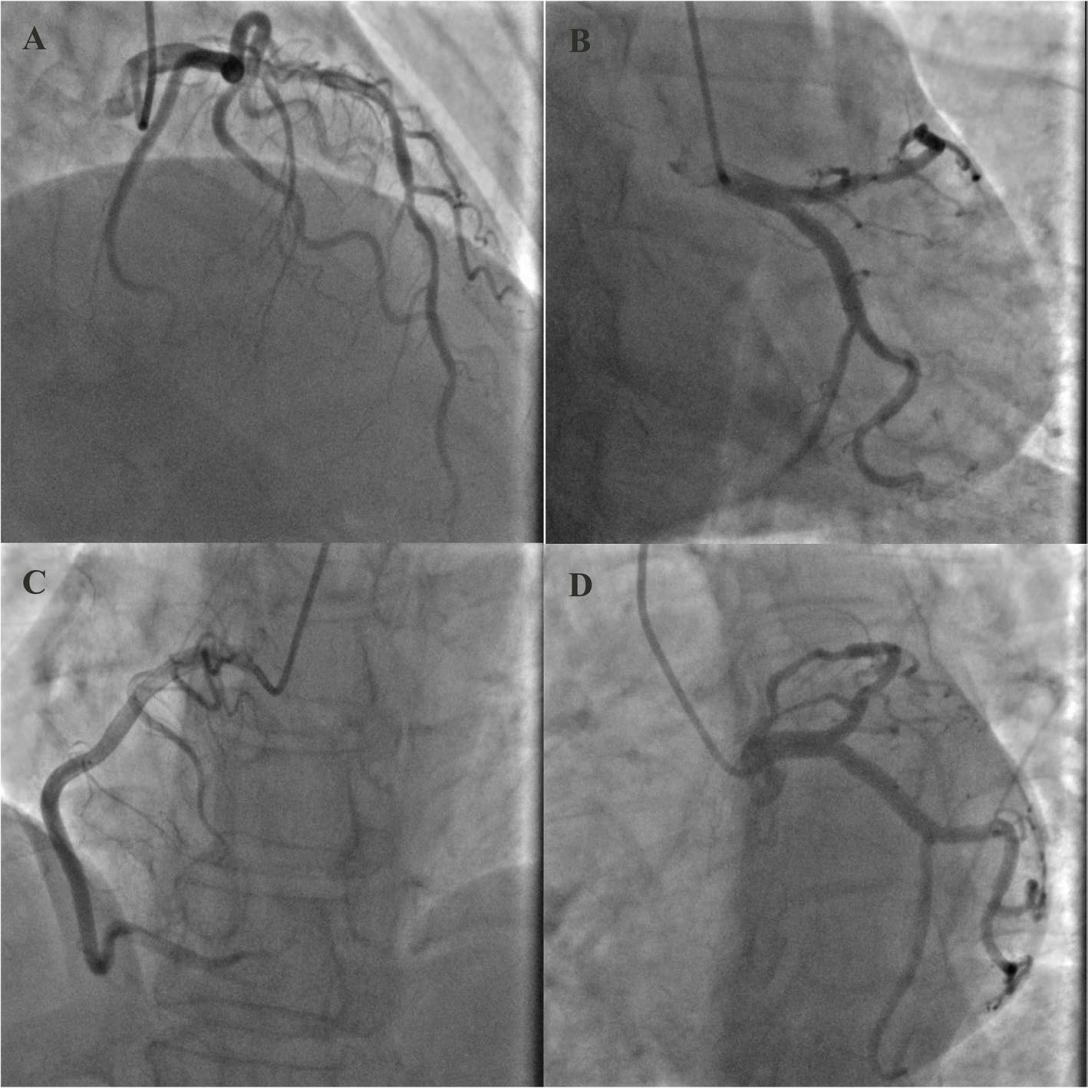
Coronary angiography revealed approximately 50%-70% stenosis in the proximal and intermediate segments of the left anterior descending artery (**A**, **D**). No stenosis was observed in the left circumflex artery (**B**, **D**) and the right coronary artery artery (**C**)

A 64-year-old woman presented to our hospital with a 3-month history of intermittent chest pain and palpitation. In addition to the hypertension and diabetes mellitus, she had an 8-year history of CLL for which she had received imatinib (420 mg daily) as initial therapy. On admission, her vital signs were normal and no remarkable abnormality was found in cardiac physical examination. The electrocardiography (ECG) showed a sinus rhythm with inverted T wave in the lead III, V1 and V2 (Fig. 1A). The laboratory results revealed a mildly elevated high-sensitive troponin (566.9 pg/ml), and her first transthoracic echocardiography examination indicated the heart chamber size, structure, and systolic function were normal except for mild mitral regurgitation (Fig. 2). Based on the symptoms and laboratory findings, the initial diagnosis was non-ST-elevation myocardial infarction (NSTEMI). The patient then underwent coronary angiography and the results showed an approximately 50%-70% stenosis in the proximal and intermediate segments of the left anterior descending artery (LAD). No stenosis was observed in the left circumflex artery (LCX) and the right coronary artery (RAC) (Fig. 3). She was then discharged with aspirin, clopidogrel, atorvastatin, and metoprolol.

**Fig. 4:**
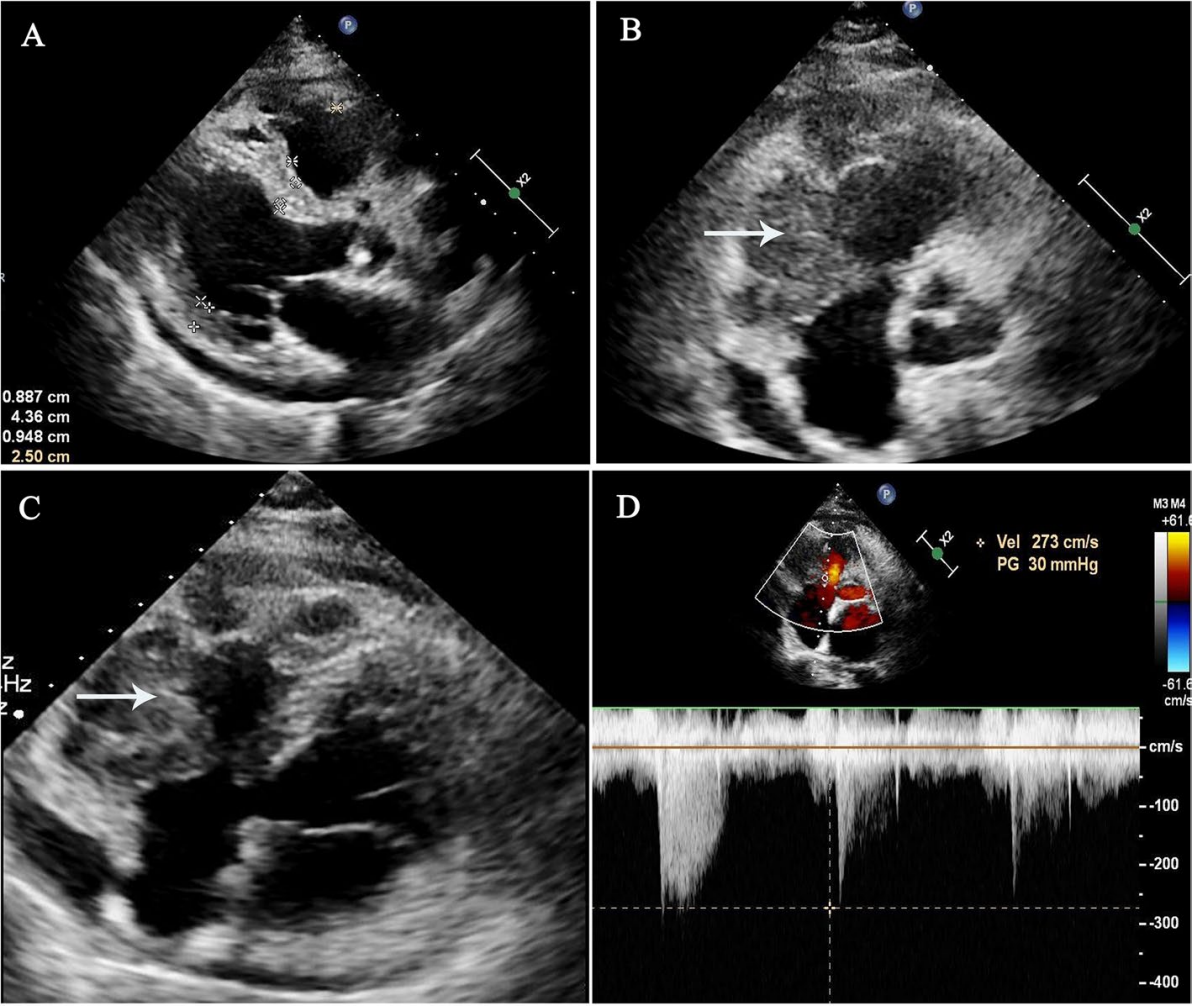
Echocardiographic findings on the second admission. **A** A mass (indicated by an arrow) measuring approximately 75.4 mm × 37.5 mm was identified in the right ventricle with infiltration and partly extending into the lateral and outflow tractof the right ventricle. **A** Parasternal long axis view indicated about 8.6 mm pericardial effusion. **B** Parasternal short axis view of the mass. **C** Apical 4 chamber view of the mass. **D** Color Doppler detected mild tricuspid regurgitation

**Fig. 5:**
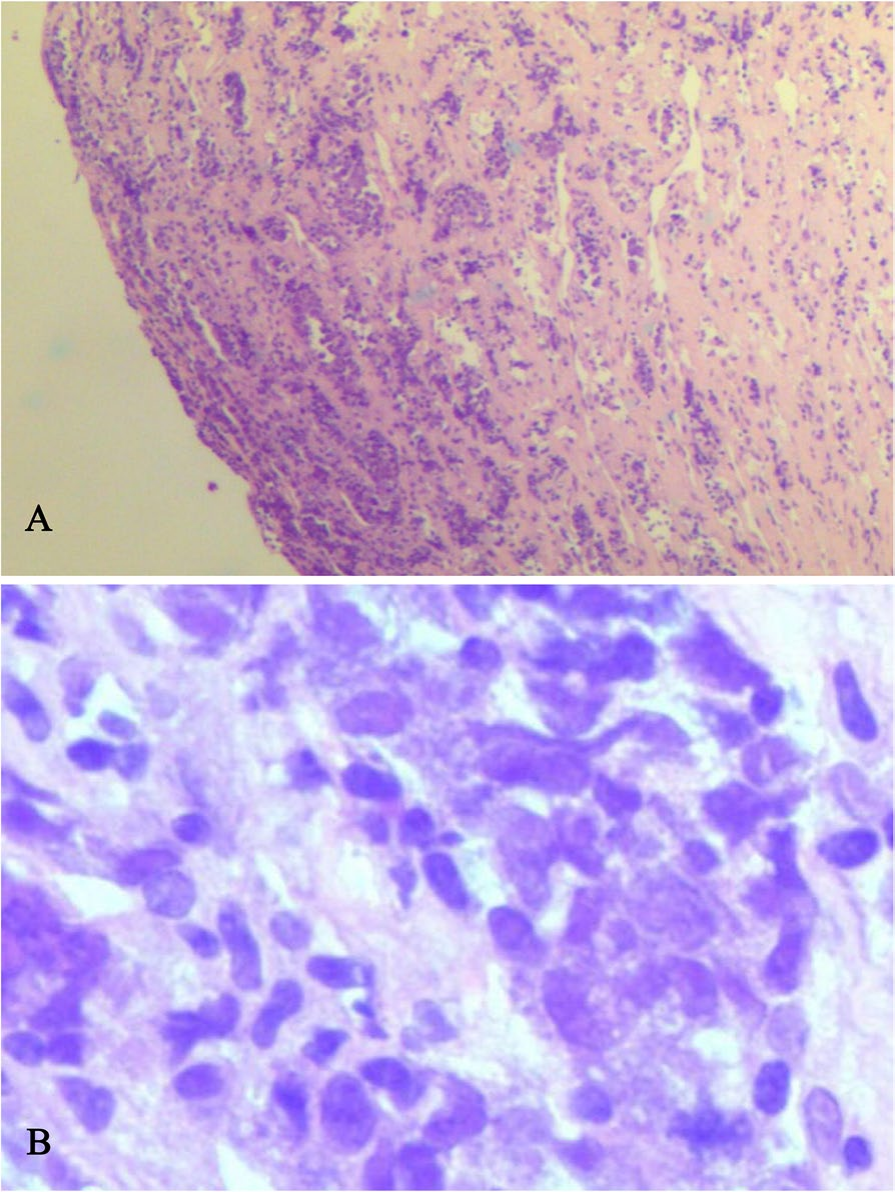
Cardiac tumor tissue, stained with hematoxylin and eosin, revealed atypical large lymphoid cells with irregular nucleoli and little cytoplasm. × 100 (**A**) and × 400 (**B**)

One month later, the patient presented to the cardiac outpatient clinic of our hospital with complaints of chest tightness and palpitation. Her ECG showed deeper inverted T wave of the inferior leads (II, III and avF), as well as leads V1 and V2 compared with the ECG obtained one month before (Fig. 1B). However, a repeated echocardiography revealed a large irregular mass, measuring about 75.4 mm × 37.5 mm. The mass was located on the lateral and the outflow tract of the right ventricle, and adhered to the anterior leaflet of the tricuspid valve, resulting in slightly restriction of the tricuspid valve opening and right ventricular wall mobility. Continuous wave Doppler (CW) showed that the peak forward tricuspid valve flow rate was 1.2 m/s, the peak pressure gradient was 6 mmHg, and the mean gradient was 4 mmHg. Color Doppler detected a mild tricuspid regurgitation, with the velocity being 2.7 m/s and the regurgitation pressure gradient being 30 mmHg. Besides, moderate pericardial effusion was observed (Fig. 4 and Supplementary Video 1). In order to identify the character of the mass the patient underwent contrast enhanced echocardiography. After injection of the contrast agent, the mass was rapidly filled with echocardiographic contrast, indicating substantial vascularization which could help exclude the thrombus. As showed in Supplementary Video 2, after clearing the microbubbles using transient high-mechanical index flash imaging, rapid contrast re-filling in the mass was observed suggesting a high extend of vascularization of the mass, which is the typical feature of malignant tumors. Then the patient underwent biopsy of the cardiac mass and the results revealed diffuse large B-cell lymphoma with dual expression of *myelocytomatosis viral oncogene (MYC)* and *B-cell lymphoma-2 (BCL2)* (Fig. 5). The chest and abdomen CT scan indicated that there was no evidence a mass elsewhere. The patient was diagnosed with Richter's syndrome and then referred to the hematology department for further chemotherapy. Until now, the patient has completed 2 times of chemotherapy with CHOP (cyclophosphamide, doxorubicin, vincristine, and prednisone) plus venetoclax and the cardiac mass is getting smaller. At present, the patient is still under follow-up.

## Discussion and conclusions

RS is a life-threatening complication of CLL, with a median survival of about 10 months. It may involve extranodal sites including gastrointestinal tract, lungs, kidneys, skin, and heart. We reported a case of rapid development of cardiac diffuse large B-cell lymphoma in a patient with preexisting CLL.

The RS transformation process is insidious, which is often challenging to diagnose and can be missed occasionally. Depending on the location, they can present with various clinical manifestations, such as chest pain, dyspnea, syncope, heart failure, pericardial effusion, valvular dysfunction, and conduction abnormalities [4]. In this case, the patient complained of chest pain and palpitation with elevated high-sensitive troponin on her first admission. However, physical examination, echocardiography, and coronary angiography revealed no significant abnormalities. It is surprising that the reexamination of echocardiography performed one month later found a large and irregular cardiac mass. This cardiac mass developed rapidly and invaded the myocardium, so it is likely to be a malignant tumor with a poor prognosis.

Accurate differentiation of cardiac mass is often difficult and an erroneous diagnosis may lead to unnecessary surgery or inappropriate anticoagulation. The advance of the modern imaging tools, such as contrast-enhanced ultrasound imaging, computed tomography (CT), and cardiac magnetic resonance imaging (MRI), increases the likelihood of preoperative diagnosis. However, these imaging modalities have their own drawbacks, such as radiation damage of the cardiac CT, as well as high technical requirements and expensive feature of cardiac MRI. More recently, contrast-enhanced echocardiography has been shown to be useful in distinguishing different types of cardiac masses, including thrombus, benign and malignant tumors. Takeuchi et al*.* found that contrast echocardiography could help detect the intracardiac thrombus in experimental animals because a thrombus retains its low echogenicity during chamber opacification [5]. It is well known that most malignant tumors have abundant neovascularization to supply the rapidly growing tumor cells, whereas the benign tumors, such as fibroma and myxomas, generally have poor blood supply and thrombi are avascular. To differentiate a thrombus from an intracardiac tumor, real-time imaging of very low mechanical index (MI) imaging with high MI flash should be used [6]. To our knowledge, few reports have reported contrast-enhanced echocardiography of cardiac lymphoma. In our case, myocardial contrast echocardiography revealed a large multilobulated mass measuring about 75.4 × 37.5 mm, occupying almost the entire right ventricle and infiltrating the free wall of right ventricle.

The contrast agent quickly entered the mass several cardiac cycles after the injection of the contrast agent and the contrast enhancement of the mass was higher than that of the normal myocardium. Combined with the patient's prior CLL, the appearance of the echocardiography suggested a cardiac lymphoma. Histological examination confirmed the diagnosis of cardiac diffuse large B-cell lymphoma. There is no standard treatment strategy for the RS transformation. Chemotherapy, chemoimmunotherapy, and stem cell transplantation are several options, determined by histological type and disease extension [7]. In a study of 74,116 patients with CLL**/**SLL, 530 patients developed RS transformation during follow up. The median age of RS diagnosis was 66 years and the median time from the diagnosis of CLL/SLL to the development of RS was approximately 4 years. RS patients had a poor outcome with a median survival of 10 months [8].

In summary, we described herein a case of a patient with cardiac presentation of Richter's transformation. Given the poor prognosis of RS, every effort should be made to diagnose these patients as early as possible. Our case also provides some experience in the diagnosis of cardiac lymphoma by contrast-enhanced echocardiography.

### Supplementary Information

**Additional file 1. **

**Additional file 2. **

## Data Availability

The data presented in this study are available on reasonable request from the corresponding author.

## Abbreviations

RS: Richter's syndrome
CLL: Chronic lymphocytic leukemia
SLL: Small lymphocytic lymphoma
DLBCL: Diffuse large B-cell lymphoma
HL: Hodgkin's lymphoma
ECG: Electrocardiography
NSTEMI: Non-ST-elevation myocardial infarction
LAD: Left anterior descending artery
LCX: Left circumflex artery
RAC: Right coronary artery
CW: Continuous wave Doppler
MYC: Myelocytomatosis viral oncogene
BCL2: B-cell lymphoma-2
CHOP: Cyclophosphamide, doxorubicin, vincristine, and prednisone
CT: Computed tomography
MRI: Magnetic resonance imaging
MI: Mechanical index
